# Generation of a Non-Transgenic Genetically Improved Yeast Strain for Wine Production from Nitrogen-Deficient Musts

**DOI:** 10.3390/microorganisms8081194

**Published:** 2020-08-06

**Authors:** Eduardo I. Kessi-Pérez, Jennifer Molinet, Verónica García, Omayra Aguilera, Fernanda Cepeda, María Eugenia López, Santiago Sari, Raúl Cuello, Iván Ciklic, María Cecilia Rojo, Mariana Combina, Cristián Araneda, Claudio Martínez

**Affiliations:** 1 Departamento de Ciencia y Tecnología de los Alimentos, Universidad de Santiago de Chile (USACH), Santiago 9170022, Chile; eikp11@gmail.com (E.I.K.-P.); jennifer.molinet@usach.cl (J.M.); 2 Centro de Estudios en Ciencia y Tecnología de Alimentos (CECTA), Universidad de Santiago de Chile (USACH), Santiago 9170022, Chile; veronica.garcia@usach.cl (V.G.); omayra_aguilera@hotmail.com (O.A.); 3 Laboratorio de Genética y Biotecnología en Acuicultura, Departamento de Producción Animal, Facultad de Ciencias Agronómicas, Universidad de Chile, Avenida Santa Rosa 11315, La Pintana, Santiago 8820808, Chile; fernandacepedalvarez@gmail.com (F.C.); me.lopez.dinamarca@gmail.com (M.E.L.); craraned@uchile.cl (C.A.); 4 Estación Experimental Agropecuaria Mendoza, Instituto Nacional de Tecnología Agropecuaria (INTA), San Martin 3853, 5507 Luján de Cuyo, Mendoza M5507EVY, Argentina; sari.santiago@inta.gob.ar (S.S.); cuello.raul@inta.gob.ar (R.C.); ciklic.ivan@inta.gob.ar (I.C.); rojo.cecilia@inta.gob.ar (M.C.R.); combina.mariana@inta.gob.ar (M.C.); 5 Consejo Nacional de Investigaciones Científicas y Tecnológicas (CONICET), Av. Rivadavia 1917, Ciudad Autónoma de Buenos Aires C1033AAJ, Argentina

**Keywords:** *Saccharomyces cerevisiae*, nitrogen consumption, genetic improvement, microsatellites, wine production

## Abstract

The yeast *Saccharomyces cerevisiae* is the main species responsible for the process that involves the transformation of grape must into wine, with the initial nitrogen in the grape must being vital for it. One of the main problems in the wine industry is the deficiency of nitrogen sources in the grape must, leading to stuck or sluggish fermentations, and generating economic losses. In this scenario, an alternative is the isolation or generation of yeast strains with low nitrogen requirements for fermentation. In the present study, we carry out a genetic improvement program using as a base population a group of 70 strains isolated from winemaking environments mainly in Chile and Argentina (F_0_), making from it a first and second filial generation (F_1_ and F_2_, respectively) based in different families and hybrids. It was found that the trait under study has a high heritability, obtaining in the F_2_ population strains that consume a minor proportion of the nitrogen sources present in the must. Among these improved strains, strain “686” specially showed a marked drop in the nitrogen consumption, without losing fermentative performance, in synthetic grape must at laboratory level. When using this improved strain to produce wine from a natural grape must (supplemented and non-supplemented with ammonium) at pilot scale under wine cellar conditions, a similar fermentative capacity was obtained between this strain and a widely used commercial strain (EC1118). However, when fermented in a non-supplemented must, improved strain “686” showed the presence of a marked floral aroma absent for EC1118 strain, this difference being probably a direct consequence of its different pattern in amino acid consumption. The combination of the capacity of improved strain “686” to ferment without nitrogen addition and produce floral aromas may be of commercial interest for the wine industry.

## 1. Introduction

*Saccharomyces cerevisiae* is a yeast species of industrial relevance given its role in the production of several alcoholic beverages, being the main species responsible for the fermentation of grape must in wine production [1]. In general terms, wine fermentation is a complex microbiological process where *S. cerevisiae* overcome its competitors transforming the sugar (glucose and fructose) into ethanol [2], contributing with alcoholic degree, aromas and flavours to the produced wines [3,4]. Additionally, the grape must is a challenging environment for yeast cells, where they face up ethanol toxicity, high osmotic pressures (20% of sugar concentration), low pH (between 2 and 3), high sulphite levels and, most importantly, limited availability of nitrogen [1,5].

The amount of initial nitrogen in the grape must is vital for the process of fermentation, being the second most consumed nutrient by yeast after carbon sources (glucose and fructose); however, while carbon sources tend to be found in excess, nitrogen sources could be in limiting concentrations [6]. Nitrogen could be present in grape must as amino acids, ammonium, peptides, urea, polyamines, amines and proteins, amongst others [7]. However, they are not all assimilated in the same way by *S. cerevisiae*. The most consumed nitrogen sources are ammonium and amino acids, and its amounts initially present in the grape must are collectively known as Yeast Assimilable Nitrogen (YAN). However, yeast growth rate is not only associated with the amount of nitrogen available but also with the quality of the nitrogen source [5,8]. Thus, nitrogen sources like ammonium, glutamine, glutamate and asparagine sustain high growth rates and therefore are considered as preferred, whereas others like proline, allantoin and urea are considered as non-preferred nitrogen sources because they allow slow growth rates [9]. Generally, ammonium is preferentially used by yeast and may occasionally be sufficient to allow their growth [10]. This nitrogen sources preferences is a consequence of a tight metabolic regulation system, where a couple of pathways interplay and overlap [11,12], with major changes in nitrogen consumption rate and the corresponding transcriptional activity associated to this trait occurring at the beginning of the fermentation [13].

One important problem for the wine industry is the deficiency of nitrogen sources in the grape must. These deficiencies produce stuck or sluggish fermentations, causing economic losses for the industry [10], which is explained by the fact that nitrogen sources are important factors regulating the biomass content during fermentation, thus directly impacting fermentation rate [14]. Currently, there are two solutions at the industrial level to minimize this effect: the first involves vineyard practices (e.g., the use of fertilizers), and the second is the use of nitrogen sources to supplement the must. However, the first solution is sometimes insufficient to provide the necessary amounts of nitrogen for the yeast and may results in the loss of grape colour and a decrease in the concentration of soluble solids [15].

One of the most common and efficient oenological practices to cover the low nitrogen contents is the addition of ammonium salts to the must, such as ammonium phosphate [16]. The suggested amount of ammonium phosphate to be added to the must for fermentation surpasses 140 mg/L [17]. However, these quantities are oftentimes insufficient to prevent sluggish or incomplete fermentations and many wine-producing companies make indiscriminate and excessive use of these inorganic compounds, leaving large quantities of residual nitrogen in the wine [16]. Wines with large quantities of residual nitrogen may result in the production of unwanted compounds such as hydrogen sulphide, that results from an imbalance is sulphur metabolism and produces bad odour, and ethyl carbamate (EC), a carcinogen produced from the reaction between urea and ethanol [18].

To avoid the effects derived from nitrogen supplementation, it has been necessary to find alternatives, for example, the isolation or generation of yeast strains with low nitrogen requirements for fermentation. This is possible to obtain given that the differences observed in nitrogen consumption by *S. cerevisiae* are not only a consequence of the fermentation conditions, but also of yeast genetics [6]. In this context, several researches have shown strain-dependent nitrogen requirements of *S. cerevisiae* in terms of nitrogen, i.e., different yeast strains have different capacities to grow and perform the fermentation process in nitrogen-limited musts due to different necessities of nitrogen [19,20,21,22].

In general, a large portion of the globally commercialized strains result from studies on the selection of strains naturally present in different ecosystems [23,24,25]. An example is the commercial yeast T73 isolated by [24] in the Mediterranean area of Alicante (Spain). Likewise, people form our laboratory has also collected and evaluated native wine yeasts at the experimental, pilot and industrial levels, one of which is currently commercialized globally (Fermicru XL strain, DSM Food Specialties). These studies indirectly use the large genetic variability naturally present in wild wine yeast populations and suggest the existence of a broad reservoir of genes and alleles that may be relevant for the improvement of strains of industrial interest.

Instead of just isolate and select, one way of obtaining improved yeast is the production of genetically modified microorganisms. However, this strategy has met with limited commercial success, with only two commercialized strains to date: one of these allows simultaneous alcoholic and malolactic fermentation [26], and the other results in a better metabolization of urea [27]. These applications were aimed at modifying one or a few genes thus limiting the applicability of the strategy due to the complexity of the traits involved and the restrictions posed by many countries on the use of genetically modified microorganisms through genetic engineering.

An alternative genetic modification strategy involving the combination of complete genomes is hybridization, which does not face the same legal restrictions. Marullo et al. proposed a model to obtain improved industrial yeast using strategies based on crosses, whereby cell-spore crosses resulted in improved yeast for fermentative parameters [28,29]. Currently, hybridization remains an efficient way to obtain improved yeast for industrial processes [30,31,32] but, as far as we know, there is no intra or interspecific hybridization programs focused on the consumption of nitrogen sources. Nonetheless, these kind of studies, in addition to studies focused in searching the genetic basis of nitrogen consumption and utilization [20,21,33,34,35,36,37], confirm that many traits of oenological interest are polygenic and, along with the high phenotypic variability in wine yeasts, support the use of these strategies for yeast improvement. However, this approach for genetic improvement have been carried out using only commercial strains, restricting the genetic variability found in the parental strains.

In the present study, we carry out a genetic improvement program using as a base population a group of 70 strains isolated from winemaking environments mainly in Chile and Argentina, mimicking a breeding strategy commonly used in plants and animals but not in yeast. It was found that the trait under study (YAN consumption) has a high heritability, obtaining strains that consume a minor proportion of the nitrogen sources present in the must. Among these improved strains, strain “686” specially showed a marked drop in nitrogen consumption without losing fermentative performance. When using this improved strain to produce wine from a non-supplemented natural grape must under wine cellar conditions, this strain showed a similar fermentative capacity with strain EC1118, and the unexpected presence of a marked floral aroma absent from this commercial strain. These characteristics of improved strain “686” may be of commercial interest for the wine industry.

## 2. Materials and Methods

### 2.1. Yeast Populations and Mating Design

To perform the genetic improvement program, we used as a base population a group of 70 strains isolated from winemaking environments (named as F_0_): 62 from Chile (Cauquenes, Curicó and Santiago), seven from Argentina (Mendoza) and one from France (Champagne) (Table S1). From this F_0_ population, a hierarchical mating strategy was established, in which each yeast strain was randomly assigned as “male” or “female”, and then “male” yeasts were crossed with different “female” yeasts, generating a first filial (F_1_) generation. To achieve this, sporulation and hybrid formation were performed as previously described [38,39], and hybrids were molecularly confirmed by interdelta fingerprinting [40] using the primers described by Xufre et al. [41].

Then, the 15 “male” yeasts and the 30 “female” yeasts with lowest breeding value for YAN were selected as breeders (one “male” mated with two “females”) to produce the second filial (F_2_) generation. Sporulation and hybrid formation were performed as previously described [38,39], and hybrids molecular confirmation was performed by interdelta fingerprinting [40,41] and then corroborated by microsatellites (SSRs), evaluating 11 loci: YDR160W (chr IV), YPL009C (chr XVI), YLR013W (chr XII), YGL184C (chr VII), YKR014C (chr XI), YBR058C (chr II), YBL084C (chr II), YOR267C (chr XV), YBR240C (chr II), YLL049W (chr XII) and YDL132W (chr IV) [42,43,44]. These marker loci were evaluated using the software Cervus 3.0 (Fields Genetics) to reconstruct the pedigree and to calculate observed heterozygosity (H_obs_), expected heterozygosity (H_exp_) and polymorphic information content (PIC) values.

### 2.2. Microscale Fermentations with Synthetic Must

Microscale fermentations of F_1_ and F_2_ strains were carried out as previously described [21,33] with some modifications. Each strain was fermented in triplicate in wine synthetic must (SM) supplemented with a final concentration of 300 mg/L YAN (SM300), according to [45], using exactly the same proportions for each nitrogen source included (ammonium and amino acids). For each experiment, the strains were initially grown under agitation in 10 mL of SM300 overnight at 25 °C. Next, 1 × 10^6^ cells/mL were inoculated into 12 mL of SM300 (in 15 mL conical tubes) and incubated at 25 °C without agitation. Fermentations were daily weighed to calculate the CO_2_ loss until the CO_2_ lost was less than 10% of the accumulated CO_2_ lost (at this point, the fermentation was considered as ended).

At the end of the fermentation, the replicas were centrifuged at 9000× *g* for 10 min and the supernatant was collected. Then, aliquots of the fermented SM300, using a Bio-Rad HPX −87H column, were injected in a Shimadzu Prominence HPLC equipment (Shimadzu) [46]. The concentration of each amino acid was measured as previously described [47], and the consumption of each nitrogen source was estimated by obtaining the difference between the final and initial amounts of each nitrogen source after and before fermentation. The residual glucose and fructose, and the production of acetic acid, glycerol, CO_2_ and ethanol, were also estimated. All these oenological traits were recorded in grams per litre (g/L) of must, except for ethanol that was measured in alcohol by volume (% *v*/*v*). Statistical analysis of oenological parameters consisted in independent ANOVA and Tukey’s tests, which were performed using R software [48].

### 2.3. Heritability Analysis

A mixed model was used to estimate the heritability (*h*^2^) and genetic correlations (*r_G_*) among all traits, and to predict the breeding values (*a*) [49]. This general model in matrix notation was:

*Y* = *Xb* + *Za* + *Ze*, (1)

where *Y* is a vector of oenological traits, *b* is the vector of fixed effects (in this case, the geographical origin), *a* is a vector of breeding values and *e* is a vector of random residual effects. *X* is a design matrix relating *Y* with fixed effects, and *Z* is other design matrix relating *Y* with breeding values and residual effects, respectively. Univariate and bivariate analyses were carried out using a Restricted Maximum Likelihood (REML) algorithm trough the DFREML version 3.0 [50] and MTDFREML [51] programs to obtain the variance and covariance components, respectively. Phenotypic correlations were estimated from direct dataset using the base package of R [52].

### 2.4. Pilot Scale Fermentations with Natural Grape Must

Fermentations with natural grape must of improved strain “686” and commercial strain EC1118 (Lallemand) were performed in the experimental cellar from the “Instituto Nacional de Tecnología Agropecuaria” (INTA) in Mendoza (Argentina). Malbec grapes harvested from Lujan de Cuyo (Mendoza, Argentina) were crushed and the grape must was treated with 50 mg/L SO_2_ and the total acidity was corrected using tartaric acid. The grape must composition was 24 °Bx and pH 3.5. The fermentations trials were carried out in triplicate in experimental units of 16 L in two conditions: without nitrogen supplementation of the grape must and with nitrogen supplementation using Fermaid^®^ AT (Lallemand) (36 mg/L). The nitrogen supplementations were carried out at 1070 g/L of must density. Each fermentative tank was inoculated with 2 × 10^6^ cell/mL, pre-growth in 12 °Bx grape must (concentrated must 68.8 °Bx diluted with distilled water at 12 °Bx, 0.2% yeast extract, 0.4% peptone, pH 4.5) for 12 h. Fermentation monitoring was performed by daily measurement of density and temperature. At the end of alcoholic fermentation, the wines were racked, physically and chemically stabilized, bottled without filtration and stored at 18° ± 2C at the INTA wine cellar. Chemical wine parameters (residual glucose and fructose, and production of acetic acid, glycerol, CO_2_ and ethanol) and nitrogen consumption (ammonium and amino acids) were analysed by HPLC as abovementioned for microscale fermentations. Statistical analysis of oenological parameters consisted in independent ANOVA and Tukey’s tests, which were performed using R software [48].

### 2.5. Sensory Analysis

A trained panel of 7 individuals (5 males and 2 females, ages ranging from 35 to 50 years old) was convened. Wines were evaluated after 6 months of bottle aging and were analysed by descriptive analysis following previously published guidelines [53]. Two sessions were held throughout the experiment, first session was used for terminology development and attribute definition, following the basic principles of descriptive analysis. Panellists defined by consensus seven aroma attributes (overall aroma, spicy, floral, dried fruit, ripe fruit, vegetative, and reduced) and three taste attributes (bitterness, acidity and concentration), and for each of them a definition and a standard, if applicable, were provided [54]. The intensity of each attribute was assessed using a non-structured 120 mm line scale that contains two reference points separated by 12 mm of each end of the line, decoding the results manually. The second session was devoted to the formal sensory evaluation of the wines, including all the wine replicates of each treatment. During the formal evaluation session, wines were presented in transparent ISO wine glasses [55] covered with plastic lids to trap volatiles, following a complete-randomized design [56]. To minimize carry-over effects, panellists were asked to follow a sip and spit protocol, rinsing their mouth with mineral water and eating a salty cracker between samples [57]. Statistical analysis of sensory parameters consisted in independent ANOVA and LSD Fisher tests, which were performed using R software [48].

## 3. Results

### 3.1. Genetic Improvement Program to Obtain Strains that Consumes Less Nitrogen

Initially, we performed random mates of individuals of the F_0_ to generate a F_1_ population composed of 195 strains belonging to 54 full and half sib families, which were confirmed by interdelta fingerprinting. This F_1_ population was phenotyped for seven oenological traits (YAN consumption, residual glucose and fructose, and production of acetic acid, glycerol, CO_2_ and ethanol) (Table S2). Using these data, we performed heritability analyses to confirm the possibility of carrying out a genetic improvement program to obtain strains that consumes less nitrogen without losing fermentative capacities (Table 1 and Table 2).

The heritabilities obtained were very high for all the traits under study, fluctuating in average from 0.71 for residual fructose to 0.99 for YAN consumption (Table 1). Moreover, several high genetic and phenotypical correlations were observed (Table 2). In general, high negative genetic and phenotypical correlations were observed among use of sugars with the production of acetic acid, glycerol and ethanol; interestingly, the phenotypical correlation between uses of both sugars was positive and very high, while genetic correlation was negative and very high. In the other hand, the genetic correlation between production of glycerol and ethanol was negative and maximum. More importantly, there is almost no phenotypic nor genetic correlation between YAN and the other six traits, indicating that, when selected, it will not affect the other oenological variables. These results allowed us to continue with the genetic improvement program focused in YAN consumption, the trait with the highest heritability of the seven oenological parameters analysed (Table 1).

### 3.2. Generation of a F_2_ Population of Improved Strains

From the previously characterized F_1_ (Table S2), we generated a F_2_ population, selecting the 23% of F_1_ strains as breeders (intensity of selection, *i* = 1.318) based on their lowest breeding values for YAN. Therefore, from the 195 strains belonging to the F_1_ generation, the 15 “male” yeasts with lowest breeding value for YAN were mated with the 30 “female” yeasts with lowest breeding value for YAN to obtain 30 full and half sib families. Due to the difficulty of identifying F_2_ hybrids by the interdelta fingerprinting, since wild strains were homothallic and thus it was not simple to differentiate a parental strain from a hybrid, an alternative strategy based in microsatellites (SSRs) was carried out.

For this purpose, six markers loci (YDR160W, YPL009C, YLR013W, YGL184C, YKR014C and YBR058C) (Table S3) for 64 suspected hybrids coming from 10 families of the F_2_ were evaluated, resulting in 11 identified as hybrids (17.19%) and 53 not identified (82.91%). Five additional marker loci (YBL084C, YOR267C, YBR240C, YLL049W and YDL132W) (Table S3) were evaluated for the same 64 suspected hybrids, identifying 20 as hybrids (31.25%), 17 as monosporic cultures (26.56%) and 27 could not be identified (42.19%). These results, added to the statistical parameters obtained for the SSRs (Table S3), confirmed their utility in the identification of hybrid strains.

**Figure 1:**
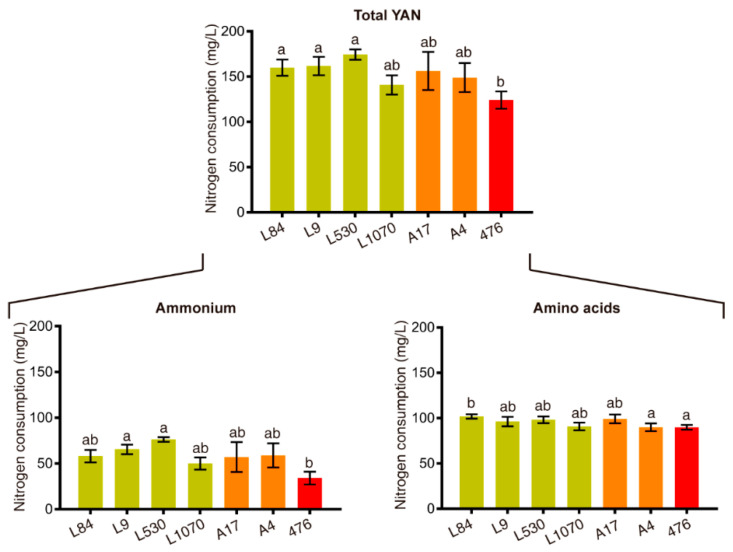
YAN consumption of improved strain “476” and their parental strains. Consumption of nitrogen sources are shown for improved strain “476” (red bar) and their respective F_1_ “parents” (A17 and A4) (orange bars) and F_0_ “grandparents” (L84, L9, L530 and L1070) (dark-yellow bars). Statistical analysis for each nitrogen source consisted in independent ANOVA followed by Tukey’s tests, with values that have a statistically significant difference (*p*-value < 0.05) having different superscript letters (a and b). Plotted values correspond to three biological replicates average, with error bars representing their standard error (mean ± SEM).

**Figure 2:**
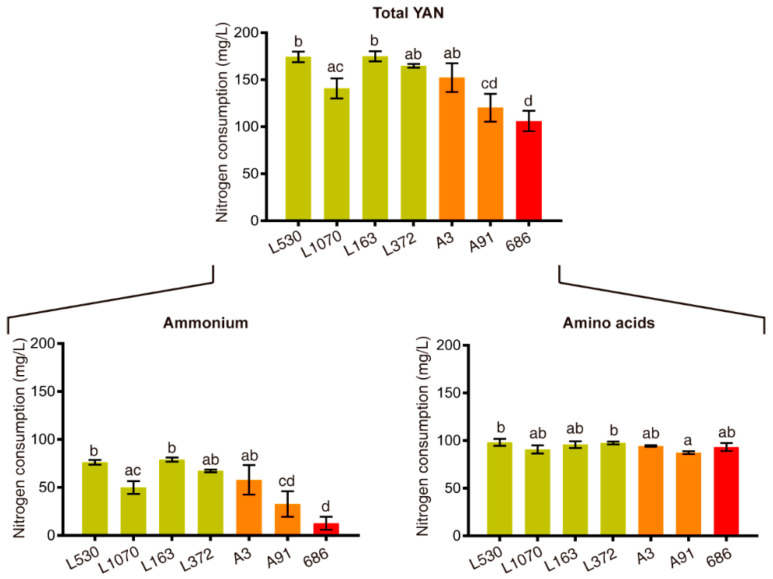
YAN consumption of improved strain “686” and their parental strains. Consumption of nitrogen sources are shown for improved strain “686” (red bar) and their respective F_1_ “parents” (A3 and A91) (orange bars) and F_0_ “grandparents” (L530, L1070, L163 and L372) (dark-yellow bars). Statistical analysis for each nitrogen source consisted in independent ANOVA followed by Tukey’s tests, with values that have a statistically significant difference (*p*-value < 0.05) having different superscript letters (a and b). Plotted values correspond to three biological replicates average, with error bars representing their standard error (mean ± SEM).

Using the above-mentioned SSRs, 51 individuals of the F_2_ population were confirmed as hybrids, belonging to 11 families with an average of three hybrids each one. Thirty-eight of these F_2_ strains, and their respective F_1_ “parents” and F_0_ “grandparents”, were fermented in SM300. Analysis of their oenological parameters showed that, while F_1_ population consume on average 187.05 ± 44.71 mg/L of YAN (Table S2), F_2_ population consume only 125.23 ± 27.69 mg/L (Table S4). Moreover, there are several F_2_ individuals that finished the alcoholic fermentation consuming a very low amount of nitrogen, even in comparison with their F_1_ parents, highlighting strains “476” and “686” (Figure 1 and Figure 2, Table 3 and Table 4, and Tables S5 and S6).

With respect to these strains, improved strain “476” consumed a smaller amount of ammonium (34.25 mg/L) compared to its parents, strains “A17” (57.06 mg/L) and “A4” (59.27 mg/L) (Figure 1 and Table S5). On the other hand, improved strain “686” required a low amount of YAN (12.76 mg/L) with respect to its parents, strains “A3” (57.94 mg/L) and “A91” (32.86 mg/L) (Figure 2 and Table S6), also consuming a greater amount of fructose than strain “A91”, thus decreasing the amount of total residual sugar (Table 4). In this sense, we obtained strains in the F_2_ population with less consumption of nitrogen sources, without losing fermentative capacities, as a product of the genetic improvement program.

### 3.3. Chemical and Sensory Analysis of Wine Produced by the Improved Strain “686”

Due to its low total YAN consumption (106.14 mg/L) (Figure 2 and Table S6), low residual sugar (0.87 g/L) (Table 4) and moderately ethanol production (12.01% *v*/*v*) (Table 4), the improved strain “686” was selected for wine production at pilot scale using a natural Malbec grape must. Grape must had an initial glucose and fructose concentrations of 113.6 and 118.8 g/L, respectively. In terms of YAN, the must without supplementation had a concentration of 80.33 mg/L, while the supplemented one had 112.27 mg/L (Table S7). Simultaneous fermentations with EC1118 commercial strain were done under the same wine cellar conditions as control treatments.

**Figure 3:**
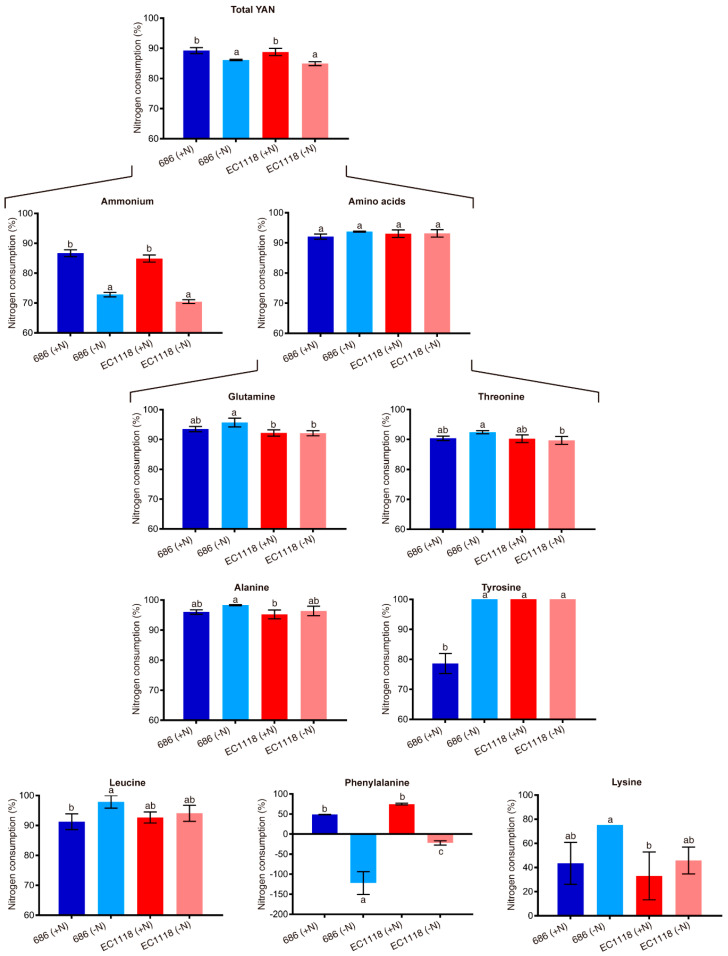
YAN consumption of improved strain “686” and commercial strain EC1118. Percentage of consumption of nitrogen sources are shown for fermentations in supplemented (+N) and non-supplemented (−N) musts. Statistical analysis for each nitrogen source consisted in independent ANOVA followed by Tukey’s tests, with values that have a statistically significant difference (*p*-value < 0.05) having different superscript letters (a, b and c). Plotted values correspond to three biological replicates average, with error bars representing their standard error (mean ± SEM).

No statistical differences were observed between chemical parameters of wines fermented with “686” and EC1118 strains neither in supplemented nor in non-supplemented must, except for a slightly higher glycerol production of improved strain “686” in non-supplemented must, suggesting that the performance of improved strain “686” was similar to this widely used commercial strain (Table S8). Regarding nitrogen consumption, both strains showed the same general pattern, reducing the consumption of total YAN and ammonium, and maintaining the consumption of total amino acids (Figure 3 and Table S9). However, the consumption levels of certain amino acids were different between strains, including glutamine, threonine, alanine, tyrosine, leucine, phenylalanine and lysine (Figure 3 and Table S9).

**Figure 4:**
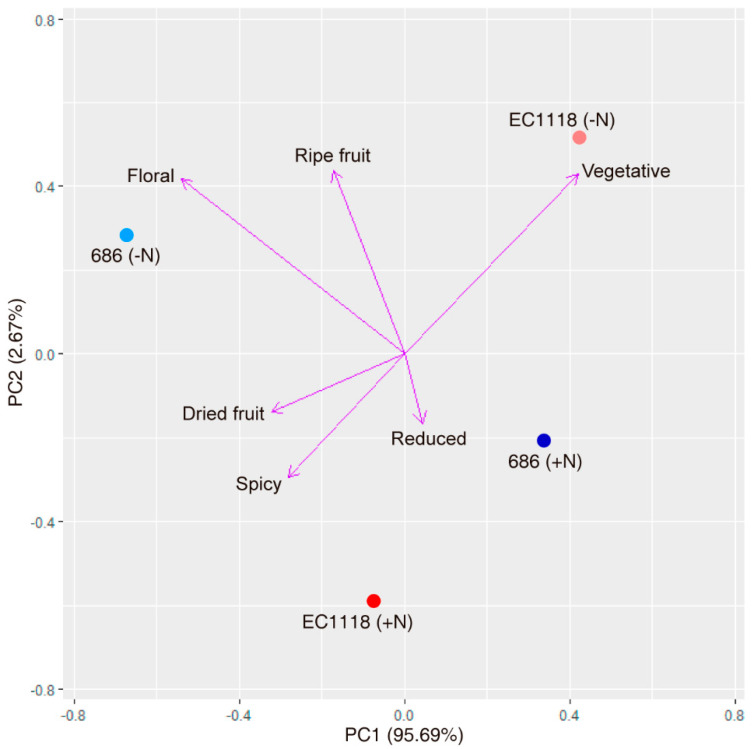
PCA plot for aroma attributes of wines produced. The relative position of the wines produced by the two strains (686 and EC1118) from supplemented (+N) and non-supplemented (−N) musts are shown for the six aroma descriptors analysed (reduced, spicy, dried fruit, ripe fruit, floral and vegetative).

Finally, a sensory analysis of the wines produced of improved strain “686” and commercial strain EC1118 was performed by a trained panel, focusing in taste and aroma characteristics. In terms of sensorial attributes, the wine produced by improved strain “686” from non-supplemented must unexpectedly stands out because of overall aroma and concentration attributes, which were perceived as statistically higher (Table 5). Additionally, this wine was associated to floral aroma and dried and mature fruit descriptors (Figure 4). These characteristics highlight the capacity of improved strain “686” not only to carry out the fermentation process without nitrogen supplementation, but also to contribute with a positive sensory impact on the final wines.

## 4. Discussion

In the present work, we performed a genetic improvement program in yeast, using a commonly used strategy in plants and animals but not in this species. In order to carry it out, an essential first step is to calculate the heritability of the phenotype to be improved in order to know the feasibility of the improvement itself. For this, we performed random crosses between F_0_ strains, thus generating families in which the phenotype was evaluated to know its heritability. In general, heritabilities were very high for all the seven oenological traits evaluated (*h*^2^ > 0.71) (Table 1). A possible explanation for these results is that these phenotypes have never been submitted to artificial selection in these yeast strains before, and therefore they keep a high percentage of genetic variance. The alternative explanation is that another environmental variation source is inflating the heritability estimations. However, other studies have reported high heritabilities values for ethanol production (0.75 < *h*^2^ < 0.80) and acetic acid production (0.80 < *h*^2^ < 0.87) [28,29]. These values obtained from a very different population support our estimation of heritabilities for these oenological traits. In terms of genetic improvement, these heritabilities indicate that artificial selection for these all seven oenological traits is highly possible and will produce a large response to selection (*R*), because it is directly proportional to the heritability value, according to the equation *R* = *h*^2^⋯*i*⋯*δ_p_*, where δ_p_ is the phenotypic standard deviation of every trait [58]. Importantly, YAN consumption showed the greater heritability (0.99) of all traits evaluated (Table 1).

Moreover, genetic correlations allow us to estimate the correlated response to selection, i.e., the genetic improvement in one trait when is selected the other trait. From this analysis, we did not observe any phenotypic or genetic correlation between YAN and the other six traits (Table 2), which indicated that this trait it will not affect the other oenological variables when selected. This idea was corroborated by the improvement program carried out, from which we obtained F_2_ strains that consumed less YAN compared to their F_1_ parents (Figure 1 and Figure 2), but without an important impact over the other oenological traits studied (Table 3 and Table 4).

Importantly, to perform the genetic improvement program it was necessary to develop a methodology based in SSRs to identify the hybrids produced in the F_2_, using 11 marker loci (Table S3). This innovation was essential, since the ability to identify hybrids is crucial for a genetic improvement program and, while in plants and animals it can often be done visually, in yeasts it must be done using molecular markers. Other works have carried out genetic improvement in yeasts but using genetically modified parental strains in order to carry out the further identification of hybrids [28,29], thus reducing the industrial potential of the improved strains obtained by being GMOs, which is not the case of the present study. The expected heterozygosity (H_exp_) (i.e., the proportion of expected heterozygous individuals in the next generation) is a measure of the genetic diversity of a population [59]. Values of H_exp_ ≤ 0.5 are not useful for large-scale analysis because the population will present more homozygous individuals and, therefore, it will not serve to identify hybrids, which was not the case for the majority of the evaluated marker loci. On the other hand, the polymorphic information content (PIC) is a measure of the informative of each SSR and is calculated from the allelic frequency [60,61]. In this context, values of PIC > 0.5 are highly informative, which is true for almost every marker locus evaluated (Table S3).

This theoretical capacity of the SSRs was tested in the identification of hybrids of the F_2_, showing a higher percentage of detection than interdelta fingerprinting. This last technique was very useful in the identification of F_1_ hybrids, obtaining a detection percentage of 32.3%, but it decreased to 5% when used in F_2_. This decrease was due to the low polymorphism presented by the interdelta regions, which caused that the band patterns between parental strains to be equal, thus individuals could not be differentiated. This effect is a direct consequence of increasing the genetic similarity between the strains, which decreased the resolution of the technique. Consequently, and because SSRs showed greater polymorphism, their detection percentage was higher (31.25%), a value similar to the obtained in F_1_ with interdelta fingerprinting. However, continuing to more generations (F_3_, F_4_, etc.) could be hampered by the increasing genetic similarity between strains, making useless not only interdelta fingerprinting but also SSRs. Therefore, it would be necessary to find other methodological alternatives that allow the confirmation of hybrids, like the identification and validation of SNPs markers.

From the genetic improvement program, two F_2_ strains, “476” and “686”, stood out given their ability to consume a very low amount of nitrogen in comparison with their F_1_ parents (Figure 1, and Tables S5 and S6) without losing fermentative capacities (Table 3 and Table 4). This result confirms that it is possible to obtain genetically improved strains by planning the crossings to be made between F_1_ members, based on their phenotypic information related to the trait to be improved. For both improved strains, the differences in total YAN consumption were driven mostly by a reduction in ammonium consumption, which is in turn an important nutrient given its status of preferred nitrogen source [8,12,62], and also by its high proportion of YAN in SM300 (approximately 40%) [45]. Between these two improved strains, strains “686” was especially interesting due to its very low total YAN consumption (106.14 mg/L) (Figure 2 and Table S6) and residual sugar (0.87 g/L) (Table 4), and then was selected for pilot scale wine production using a natural grape must coming from Malbec grapes. It would be of great importance to know the specific genetic variants that allow these strains to perform fermentation consuming less nitrogen than its parental strains, for which other studies focused on that search are needed.

The fermentative behaviour of improved strain “686” in natural must was comparable to a widely used commercial strain (EC1118) both in conditions of nitrogen supplementation and non-supplementation. However, amino acid consumption profile was different between them (Figure 3 and Table S9). Moreover, in terms of organoleptic characteristics, wines produced by improved strain “686” from the non-supplemented must showed higher overall aroma and concentration values (Table 5). In this sense, it is interesting to note that both strains showed different profiles of consumption of aromatic amino acids tyrosine and phenylalanine (Figure 3 and Table S9), which can be linked to differences in overall aroma. Moreover, volatile metabolites are known to be derived from sugar and amino acid metabolism, including higher alcohols, esters, carbonyls, sulphur compounds and volatile fatty acids [63]. Despite the different views in the strength of the link between must YAN and volatile compounds production, with studies showing a significant correlation [18,64,65,66,67], while others a much weaker one [68,69], it seem to be clearly dependent on the strain used in each case [5].

The wine produced by this strain “686” from the must without nitrogen supplementation not only showed a higher overall aroma (Table 5), but also the presence of a marked floral and fruit aroma (Figure 4). In general, and despite published information on aroma descriptions and flavour chemistry, perceived aromas and flavours are the result not of a single compound but of specific ratios of many compounds, and then these characteristics of wine cannot be easily predicted [1]. However, in the case of floral aromas, they are known to be caused specially by monoterpenes [63]. While these molecules are traditionally considered to be linked mainly to the grape variety, recent evidence point to the capacity of some strains of *S. cerevisiae* to contribute to the floral aroma of wine by *de novo* synthesis of monoterpenes [63,70]. Interestingly, this capacity seems to be augmented in musts with higher concentrations of YAN (like the ammonium ion) [70], an idea that is linked to the fact that addition of nitrogen sources to reach 320 mg/L of YAN concentration in grape musts led to wines with higher intensities of most of the floral and fruity attributes [5]. This poses the question that if improved strain “686” is capable of produce monoterpenes in conditions of low nitrogen, and if this capacity is linked to its amino acid consumption profile. More studies focused in the production of volatile compounds by this strain and its identification by analytical analyses are necessary to clarify these points, in addition with the determination the genetic variants that allow the improved strain “686” to produce wines with the identified organoleptic characteristics, as also its evaluation in natural musts coming from other grape varieties.

## 5. Conclusions

In conclusion, a genetic improvement program to obtain yeast strains that carry out wine fermentation with less nitrogen consumption was performed, founding that this trait has a high heritability (0.99). In the F_2_ population, strains that consume a minor proportion of the nitrogen sources present in the must were obtained, highlighting improved strain “686”. This strain was used to produce wine from a natural grape must at pilot scale, showing that in non-supplemented must it has a comparable fermentative performance with the commercial strain EC1118. Also, sensory attributes as overall aroma, concentration, floral and fruit which bears a positive connotation were perceived as significantly higher in the wines produced with the improved strains. More studies are required to understand the possible connexion between the amino acid consumption profile of improved strain “686” in non-supplemented must and the sensory attributes of the wine produced by this strain, especially in terms of production of volatile compound like monoterpenes and other molecules that contribute to wine aroma.

## 6. Patents

CL 20193925: “Cepas de levadura *Saccharomyces cerevisiae* no transgénicas mejoradas genéticamente, su uso para la producción de bebidas alcohólicas a partir de mostos deficientes en nitrógeno y su método de obtención mediante un programa de mejoramiento genético intraespecífico”.

## Figures and Tables

**Table 1:** Univariated (on the diagonal), bivariated (out of the diagonal) and average heritabilities (in bold) for the seven oenological traits evaluated.

Trait	YAN	CO_2_	ACE	GLU	FRU	GLY	ETH
**YAN**	0.9707	0.9057	0.8007	0.9597	0.2909	N.D.	0.8886
**CO_2_**	0.9926	0.9247	0.8134	0.8023	0.9075	0.9998	0.8638
**ACE**	0.9995	0.9575	0.9297	0.9936	0.9999	0.9610	1.0000
**GLU**	0.9965	0.9996	0.9825	0.7278	0.0794	0.9969	0.7947
**FRU**	0.9888	0.7535	0.9998	0.0249	0.7225	1.0000	0.9375
**GLY**	0.9999	N.D.	0.9959	0.9288	0.9996	0.8627	0.0268
**ETH**	0.9999	0.9693	0.9661	0.6314	0.9858	0.3866	0.7540
**Average**	**0.9926**	**0.9184**	**0.9269**	**0.7241**	**0.7122**	**0.8678**	**0.7522**

YAN: Total YAN consumption. CO_2_: CO_2_ production. ACE: Acetic acid production. GLU: Residual glucose. FRU: Residual fructose. GLY: Glycerol production. ETH: Ethanol production. N.D.: Not determined. Univariate marked with gray background.

**Table 2:** Phenotypic (above diagonal) and genetic (below diagonal) correlations for the seven oenological traits evaluated.

Trait	YAN	CO_2_	ACE	GLU	FRU	GLY	ETH
**YAN**		−0.0456	0.0291	0.2510	−0.0016	−0.0174	−0.2551
**CO_2_**	0.0013		0.4172	−0.1330	−0.5841	N.D.	0.2406
**ACE**	0.0421	0.3714		−0.6822	−0.5950	0.8395	0.5915
**GLU**	0.3099	−0.1394	−0.6798		0.9028	−0.7310	−0.9229
**FRU**	0.2089	−0.3546	−0.5951	−0.9999		−0.0235	−0.9283
**GLY**	−0.0174	N.D.	0.8708	−0.7748	−0.3546		0.6530
**ETH**	−0.2753	0.1963	0.6018	−0.9232	−0.9347	−1.0000	

YAN: Total YAN consumption. CO_2_: CO_2_ production. ACE: Acetic acid production. GLU: Residual glucose. FRU: Residual fructose. GLY: Glycerol production. ETH: Ethanol production. N.D.: Not determined. Univariate marked with gray background.

**Table 3:** Oenological parameters improved strain “476” and their F_1_ parental strains.

Strain	Acetic Acid Produced ± SD (g/L)	Residual Glucose ± SD (g/L)	Residual Fructose ± SD (g/L)	Glycerol Produced ± SD (g/L)	Ethanol Produced ± SD (% *v*/*v*)
A17 ^α^	2.00 ± 0.20 ^a^	6.04 ± 8.64 ^a^	28.33 ± 20.42 ^a^	10.07 ± 1.42 ^a^	10.15 ± 2.05 ^a^
A4 ^α^	2.29 ± 0.06 ^a^	0.05 ± 0.08 ^a^	9.09 ± 10.74 ^a^	11.46 ± 0.66 ^a^	10.34 ± 0.81 ^a^
476 ^β^	2.30 ± 0.10 ^a^	0.00 ± 0.00 ^a^	3.52 ± 4.84 ^a^	10.15 ± 0.67 ^a^	10.92 ± 0.72 ^a^

^α^ Parental strains from F_1_ population. ^β^ Improved strain from F_2_ population. SD: Standard deviation. Statistical analysis consisted in independent ANOVA followed by Tukey’s tests, with values that have a statistically significant difference (*p*-value < 0.05) having different superscript letters (a and b).

**Table 4:** Oenological parameters improved strain “686” and their F_1_ parental strains.

Strain	Acetic Acid Produced ± SD (g/L)	Residual Glucose ± SD (g/L)	Residual Fructose ± SD (g/L)	Glycerol Produced ± SD (g/L)	Ethanol Produced ± SD (% *v*/*v*)
A3 ^α^	2.34 ± 0.07 ^a^	0.00 ± 0.00 ^a^	1.10 ± 0.47 ^a^	11.06 ± 0.66 ^a^	11.94 ± 0.58 ^a^
A91 ^α^	2.15 ± 0.01 ^b^	1.71 ± 1.87 ^a^	23.19 ± 11.54 ^b^	10.05 ± 0.87 ^a^	10.95 ± 0.99 ^a^
686 ^β^	2.41 ± 0.13 ^a^	0.00 ± 0.00 ^a^	0.87 ± 0.57 ^a^	9.45 ± 0.45 ^a^	12.01 ± 0.41 ^a^

^α^ Parental strains from F_1_ population. ^β^ Improved strain from F_2_ population. SD: Standard deviation. Statistical analysis consisted in independent ANOVA followed by Tukey’s tests, with values that have a statistically significant difference (*p*-value < 0.05) having different superscript letters (a and b).

**Table 5:** Values obtained for descriptive sensory attributes of Malbec wines at pilot scale.

Strain	Nitrogen Supplementation	Overall Aroma ± SD	Bitterness ± SD	Acidity ± SD	Concentration ± SD
686	Yes	3.82 ± 2.38 ^a^	3.97 ± 0.57 ^a^	6.07 ± 0.83 ^a^	4.48 ± 1.66 ^a^
	No	6.47 ± 2.37 ^b^	3.79 ± 0.83 ^a^	4.43 ± 1.75 ^ab^	6.57 ± 0.84 ^b^
EC1118	Yes	5.46 ± 1.06 ^ab^	1.58 ± 1.25 ^b^	3.99 ± 1.42 ^b^	4.26 ± 0.93 ^a^
	No	3.55 ± 0.99 ^a^	3.52 ± 0.66 ^a^	5.74 ± 1.78 ^ab^	4.31 ± 1.89 ^a^

SD: Standard deviation. Statistical analysis consisted in independent ANOVA followed by LSD Fisher tests, with values that have a statistically significant difference (*p*-value < 0.05) having different superscript letters (a and b).

## Supplementary Materials

The following are available online at https://www.mdpi.com/2076-2607/8/8/1194/s1, Table S1: Geographical origin of F_0_ population, Table S2: Oenological parameters of F_1_ population, Table S3: Statistical parameters for evaluated SSRs, Table S4: Oenological parameters of F_2_ population, Table S5: YAN consumption of improved strain “476” and their F_1_ and F_0_ parental strains, Table S6: YAN consumption of improved strain “686” and their F_1_ and F_0_ parental strains, Table S7: Initial YAN composition of natural grape must, Table S8: Oenological parameters of improved strain “686” and commercial strain EC1118, Table S9: YAN consumption of improved strain “686” and commercial strain EC1118.
